# Probiotic and anti-inflammatory attributes of an isolate *Lactobacillus helveticus* NS8 from Mongolian fermented koumiss

**DOI:** 10.1186/s12866-015-0525-2

**Published:** 2015-10-02

**Authors:** Jingjing Rong, Houfeng Zheng, Ming Liu, Xu Hu, Tao Wang, Xingwei Zhang, Feng Jin, Li Wang

**Affiliations:** The Affiliated Hospital, School of Medicine, Hangzhou Normal University, Hangzhou, China; Institute of Psychology, Chinese Academy of Sciences, Beijing, China; Institute of Genetics and Developmental Biology, Chinese Academy of Sciences, Beijing, China; University of the Chinese Academy of Sciences, Beijing, China

**Keywords:** *L. helveticus*, Anti-inflammatory effect, S-layer protein, Koumiss

## Abstract

**Background:**

Koumiss is a traditionally fermented mare’s milk described with health-promoting potentials for decades. However, only a few studies focused on the probiotic strains isolated from koumiss. In this study, we collected koumiss samples from Inner Mongolian pasturing area of China and selected a promising strain of *Lactobacillus helveticus*, isolate NS8, based on the survival abilities in gastrointestinal tract (GIT) and adhesion to intestinal endothelial cells *in vitro*. As the ability to positively modulate host immune response is a feature of increasing importance in measuring the probiotic potential of a bacterial strain, our study mainly focus on the immunomodulatory properties of *L. helveticus* NS8 by using *in vivo* and *ex vivo* analyses.

**Results:**

*L. helveticus* NS8 was identified by molecular-typing methods, both at genus and species levels. As a typical food niche-specific bacteria, NS8 showed a moderate survival ability in GIT environment *in vitro*. However, an excellent binding capacity to the human intestinal epithelial cells, along with significant autoaggregation and cell-surface hydrophobicity was observed. Additionally, the presence of S-layer protein was responsible for the cell surface properties of this strain. NS8 was found to be rather protective against TNBS (2,4,6-trinitrobenzene sulfonic acid)-induced murine colitis. In the meantime, co-culture with NS8 induced an increased level of secretion of anti-inflammatory cytokine IL-10 in peripheral blood mono-nuclear cells (PBMCs). Furthermore, NS8 was also able to diminish the proinflammatory effects of lipopolysaccharide (LPS) in mouse macrophage cell line RAW264.7 by inducing higher levels of IL-10. Specially, adding of the purified S-layer protein didn’t influence the production of IL-10. The specific ligand-host receptor interactions on the NS8 specific immune responses need to be learned further.

**Conclusion:**

In summary, *L. helveticus* NS8 exhibited good probiotic and particularly immunomodulatory properties, with a potential for development of functional food commercially or therapeutic adjuvant for inflammatory diseases.

**Electronic supplementary material:**

The online version of this article (doi:10.1186/s12866-015-0525-2) contains supplementary material, which is available to authorized users.

## Background

Probiotic lactobacilli have been increasingly implicated in a number of health-promoting functions [1, 2]. Quite a part of commercial probiotic lactobacilli isolated from intestinal microbiota, such as *Lactobacillus acidophilus*, *L. rhamnosus*, *L. johnsonii*, *L. paracasei*, and *L. reuteri* have been widely demonstrated to interact with gut physiological processes and confer benefits to host organisms [3, 4]. On the other hand, some *Lactobacillus* species, such as *L. delbrueckii*, *L. helveticus*, and *L. plantarum*, are traditionally involved in the production of fermented foods [5]. Unlike the commensal lactobacilli that colonize the intestinal tract, food originated *Lactobacillus* cells are ingested along with food flow during the consumption of fermented products and, consequently, may directly come in contact both with the host’s gastrointestinal mucosa and intestinal microbiota. The potential influence of food-associated lactobacilli on host’s health compared to that of intestinal lactobacilli, however, has been poorly investigated.

*Lactobacillus helveticus* strains are extensively used in manufacture of Swiss-type cheeses and Italian cheeses (Emmental, Grana Padano, Mozzarella), as well as fermented drinks [6]. Remarkably, genome sequencing had revealed that *L. helveticus* DPC4571 isolated from cheese whey shares the nearest identity (98.4 %) with the gut organism *L. acidophilus* NCFM, one of probiotic strains thoroughly investigated for probiotic functions [7]. Giving fermented food originated bacterium had its adaptive evolution in dairy environment, there is strong need to explore its probiotic properties and crosstalk with host immune system as well as intestinal bacteria. Some researches had demonstrated the efficacy of *L. helveticus* in modulating of host physiology. For instance, milk fermented with *L. helveticus* R389 decreases the growth rate of mammary tumors [8]. Furthermore, the presence of S-layer proteins was proposed to contribute to probiotic properties of *L. helveticus* strains [9]. S-layer protein of *L. helveticus* MIMLh5 was recently demonstrated to be involved in immunomodulatory effects [10].

Koumiss is a spontaneously fermented mare’s milk drink and has been traditionally popular among nomadic populations of Central Asia and Mongolian areas [11]. In 1980s, a batch of studies had reported the therapeutic potentials of koumiss on the alimentary canal’s activity, the circulatory, nervous systems and the immune system [12, 13]. However, only a few studies focused on the probiotic strains isolated from koumiss. In this study, a promising isolate NS8 was selected from fermented koumiss of Mongolian pasturing area, and identified as *L. helveticus* by genus and species specific PCR as well as 16S rRNA sequencing. The selected culture was subjected to characterization for functional and probiotic attributes. To determine the suitability of NS8 for exploitation as a probiotic, particularly for application in functional food, we studied the probiotic properties like pH and bile salt tolerance, cell surface hydrophobicity and autoaggregation, and anti-inflammatory activities. In addition, we also investigated the presence of S-layer protein and its role in promoting immunomodulatory activity in a LPS-induced mouse macrophage cell line RAW264.7. NS8 showed prominent results in inducing IL-10 expression. Because IL-10 plays a central role in downregulating inflammatory cascades, strains capable of inducing this cytokine would likely be good candidates for use in anti-inflammatory intervention. Nevertheless, substantial differences were found among probiotic strains in their capacity to induce cytokine profile [14, 15]. Recently, IL-10/IL-12 cytokine induction ratio on peripheral blood mononuclear cells (PBMCs) has also been used to distinguish the anti-inflammatory properties of probiotics [16]. We employed both *in vitro* cytokine production assay and a murine trinitrobenzene sulfonate (TNBS) model of acute colitis *in vivo*. In this paper, we describe the various probiotic and functional properties of *L. helveticus* NS8 and could provide important insights into the health-modulating potential of this strain.

## Results

### Screening and genetic identification of NS8

**Fig. 1:**
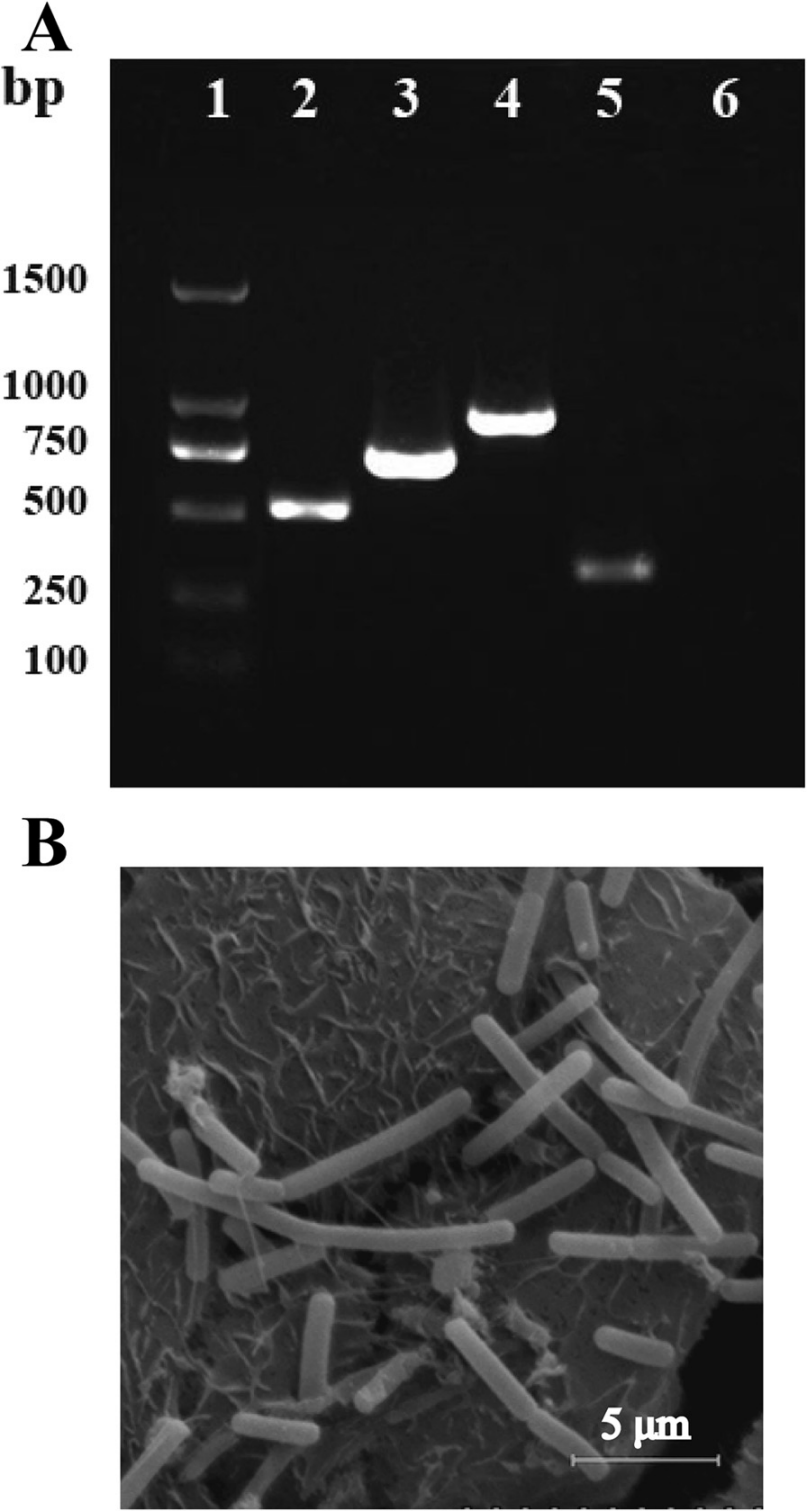
Molecular identification of *L. helveticus* NS8 isolate. **a**. PCR typing of NS8 isolate by using species-specific *pepC*/*pepN*/*htrA* primers (lane 2, *pepC*; lane 3, *pepN*; lane 4, *htrA*) and *L. helveticus* specific primers for amplification of partial 326 bp *slpH* gene (lane 5). There is no PCR product obtained by using primers targeted against *L. acidophilus slpA* (lane 6). Lane 1, DNA ladder. **b**. Morphology of NS8 under scanning electron microscope. NS8 are rod-shaped bacteria with high adhesion capacity to Caco-2 cells

In total, 12 typical colonies of lactic acid bacteria isolates were obtained from fermented koumiss based on morphological characteristics after Gram’s staining and catalase negative reaction. By testing the survival abilities in GIT environment and adherent potential to intestinal mucosa *in vitro*, only strain NS8 was selected as candidate for probiotic, which showed higher tolerance to low pH (70 % survival rate at pH 2) and toxic bile salts (65 % survival rate in 0.3 % bile). Then we identified this strain as *L. helveticus* NS8 by performing 16S rRNA gene sequencing (Genbank accession No. JQ013296.1) and BLAST search. Considering the wide inter-strain variability of *L. helveticus* and difficult discrimination between *L. helveticus* and closely related species, such as *L. acidophilus* and *L. delbrueckii*, *L. helveticus* specific primer pairs targeting the sequences of aminopeptidases C and N, and a trypsin-like serine protease gene (*pepC*, *pepN*, *htrA*) were applied in a species specific PCR assay [17]. The PCR fragments obtained had sizes of about 500, 700 and 900 bp respectively (Fig. 1a), which were in good accordance with the expected sizes.

### Surface properties of NS8

To investigate the surface properties of NS8, the isolate as well as food-originated probiotic strains *L. acidophilus*1.2 [18] and *L. plantarum* TH1 [19] (provided by Prof. XG Luo, Tianjin University of Science and Technology) were subjected to an array of tests. As shown in Table 1, the adhesion ratio of NS8 isolate with Caco-2 cell culture was estimated to be 18.03 %, which was significantly higher than the other probiotic strains with values of 4.58 % of *L. acidophilus* 1.2 and 3.34 % of *L. plantarum* TH1 respectively. Strong adhesion of NS8 to Caco-2 cells could also be clearly seen in the SEM micrographs of Fig. 1b. Besides, NS8 exhibited the highest cellular autoaggregation (79.5 %) compared with *L. acidophilus*1.2 (42.3 %) and *L. plantarum* TH1 (22.6 %). The autoaggregating phenotype of NS8 was so strong that over half bacteria have formed a precipitate in 1 h (see Additional file 1), while other bacterial suspensions showed constant turbidity with little precipitate. With a similar trend, the hydrophobic values obtained for NS8 in the presence of xylene was significantly higher than the other two strains. Adhesion to chloroform (electron acceptor) and ethyl acetate (electron donor) was also tested to assess the Lewis acid-base characteristics of the bacterial cell surfaces (see Additional file 2).

**Table 1:** Cell surface properties of *L. helveticus* NS8 and other probiotic cultures

	Cell surface properties		
Bacteria	Adhesion (%)	Aggregation (%)	Hydrophobicity (%)
*L. helveticus* NS8	18.0 ± 1.3 a	78.8 ± 2.4	56.8 ± 1.0
*L. acidophilus* 1.2	4.6 ± 0.6	41.9 ± 3.1	33.8 ± 2.1
*L. plantarum* TH1	3.1 ± 0.1	22.5 ± 2.7	32.1 ± 0.5

^a^All the values are shown as mean ± SD of triplicate experiments

### Influence of S-layer protein on cell surface properties

Two sets of primers targeted against S-layer gene of *L. helveticus* (*slpH*) and *L. acidophilus* (*slpA*) respectively were designed, according to the genome sequences of *L. helveticus* DPC4571 (GenBank accession No: CP000517.1) and *L. acidophilus* NCFM (GenBank accession No: NC006814.3). Fig. 1a showed the amplification of an expected PCR product of size 300 bp when assayed using *slpH* primers, but no product by using *slpA* primers.

**Fig. 2:**
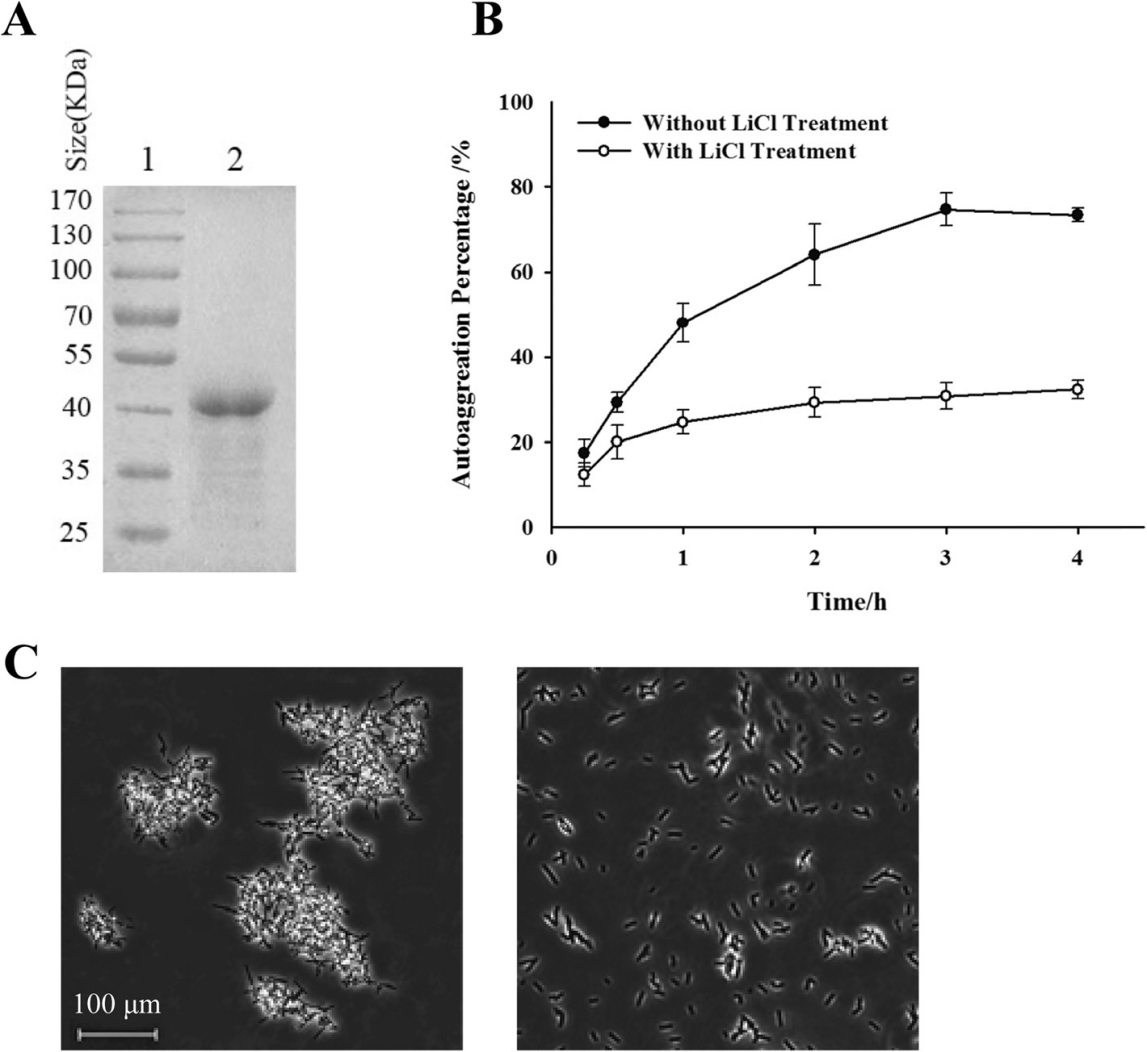
Influence of S-layer protein on the cell surface properties of *L. helveticus* NS8. **a**. SDS-PAGE profile with Coomassie blue staining of extracted S-layer protein by LiCl treatment, indicating an approximate mass value of 42 kDa. Lane 1, low molecular weight protein standards; lane 2, 5 μg of purified protein was loaded on the gel. **b**. Comparison of the autoaggregation ability of NS8 before (●) and after removal of their surface layer proteins (○). Error bars represent standard deviations (SD) of the mean values of results from three replicate experiments. **c**. Bacterial micrographs of NS8, showing the morphology difference between autoaggregated cells (*left*) and those cells after removal of S-layer proteins (*right*). Magnification, × 100

For extraction the S-layer protein from NS8 bacteria, a generally employed method for the removal of S-layer proteins from the cell surfaces, LiCl extraction, was applied for NS8. SDS-PAGE revealed the molecular mass of the protein is approximately 42 kDa, basically in line with those characterized S-layer proteins (Fig. 2a). We verified whether the protocol based on LiCl washes efficiently removed most of the S-layer proteins from the surface of *L. helveticus* cells. After S-layer removal, autoaggregation ability of NS8 reduced dramatically from 70 to 32 % (Fig. 2b). The bacterial morphology also changed from appearance of cluster into separated cells under microscope (Fig. 2c). These results indicated that the presence of S-layer protein contributed to the cell surface properties of NS8.

### Anti-inflammatory attributes

#### Colitis protective ability in vivo

**Fig. 3:**
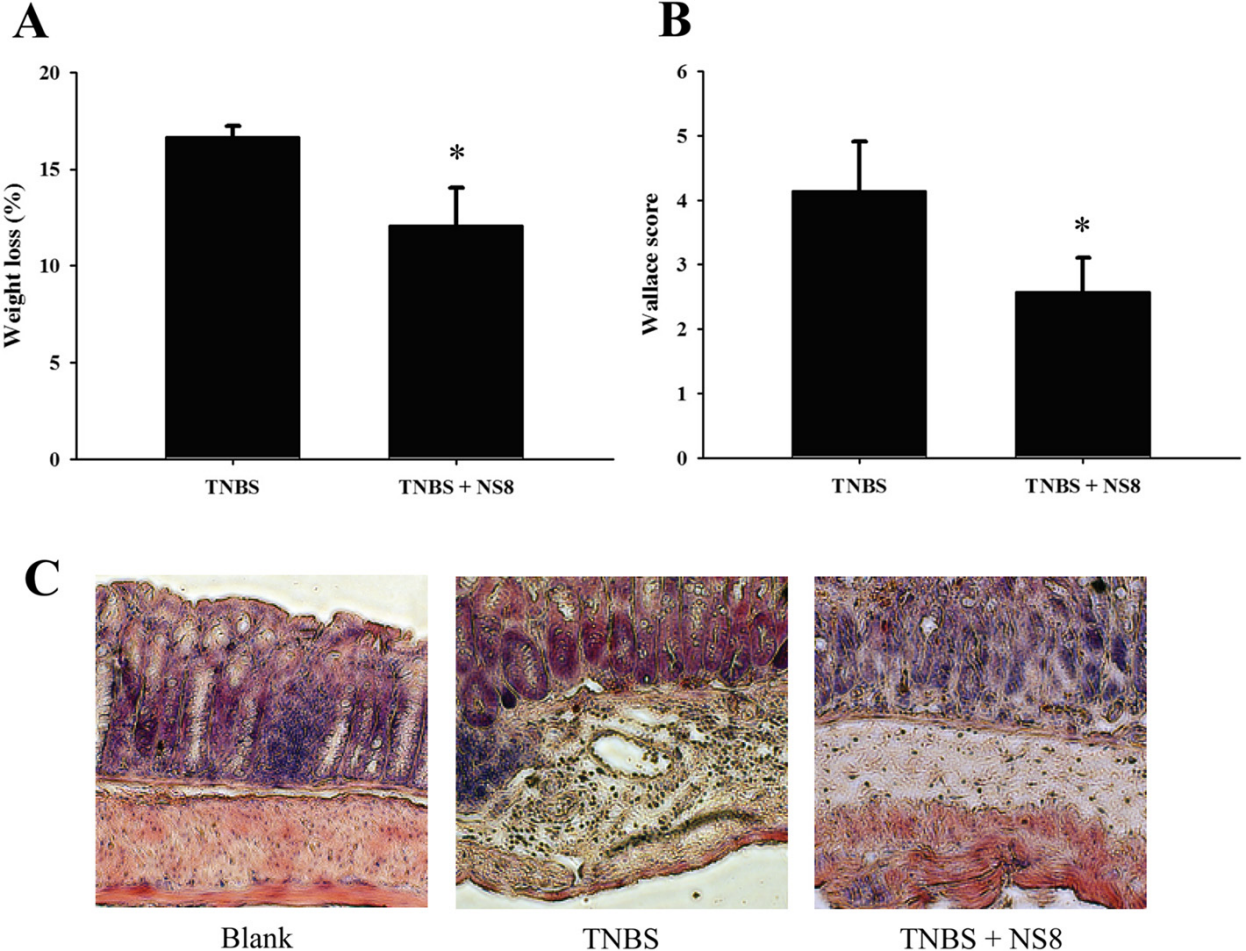
Anti-inflammatory effect of *L. helveticus* NS8 on acute colitis induced in BALB/c mice by intrarectal administration of TNBS. **a**. Weight variation between day 5 (TNBS administration) and day 7 (death). **b**. Wallace inflammation scores. Results are means ± SEM of one representative experiment (ten mice per group). Significant *P* < 0.05 (*), as compared with the TNBS control group. **c**. Histological sections of colonic tissues of BALB/c mice. Left, blank control group receiving ethanol 50 %; Middle, TNBS-treated group receiving no NS8; Right, TNBS-treated mice gavaged with NS8. Magnification, ×100

To evaluate the immunomodulatory potential of NS8, we compared the development of TNBS-induced colitis in mice that were treated, or not, with NS8. We observed that weight loss was significantly reduced in mice receiving NS8 (12.1 %) as compared with mice not received bacteria (16.7 %, Fig. 3a). Characteristic features of colitis were observed 2 days after administration of TNBS in the mice receiving no bacteria, leading to a Wallace score of 4.2 ± 0.8 (Fig. 3b), which corresponds to several areas of ulceration accompanied by intestinal wall thickening. Histological analysis performed on these mice revealed large areas of ulceration with inflammatory infiltrates (Fig. 3c). In contrast, mice that had received NS8 bacteria displayed significantly less severe lesions. Necrotic lesions were observed in only three mouse of ten, whereas the others suffered mildly from hyperemia and thickening of the intestinal wall, leading to a Wallace score of 2.6 ± 0.5 (Fig. 3b, c).

#### Cytokine stimulation in human PBMCs

**Fig. 4:**
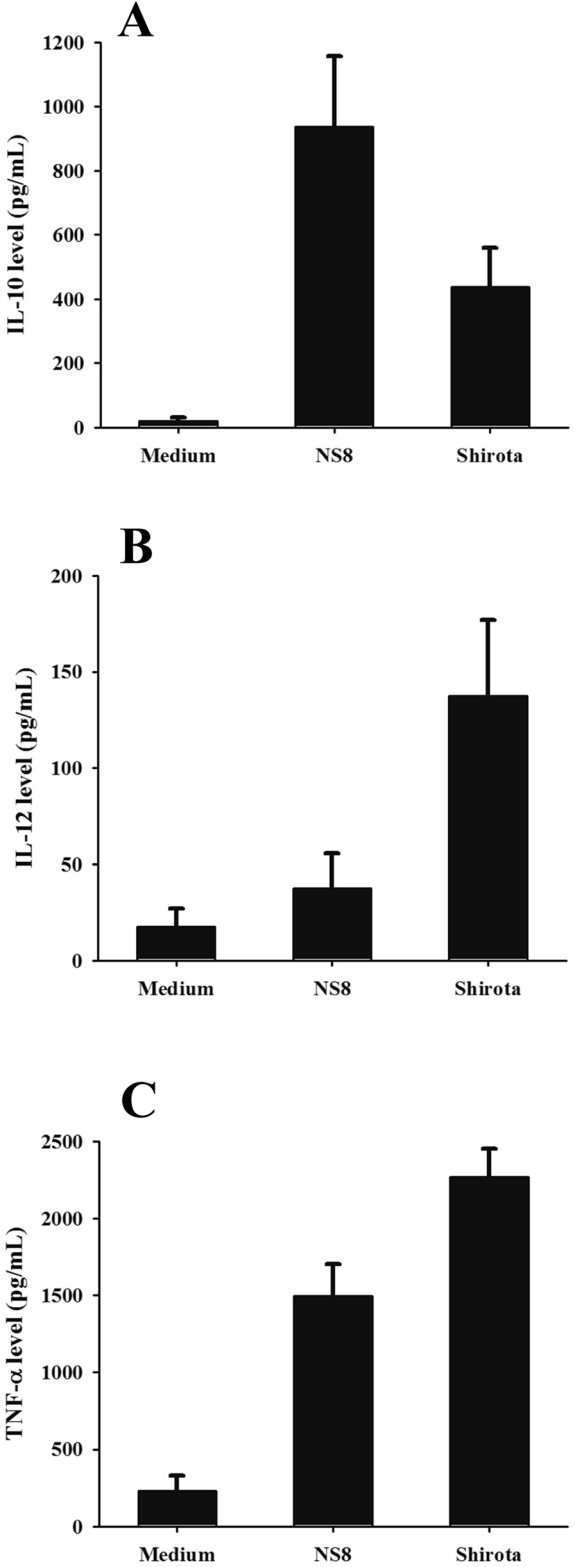
Cytokine response of human PBMCs to stimulation with *L. helveticus* NS8 and *L. casei* Shirota strains. Cell supernatant were collected after 24 h co-incubation with bacteria (bacteria: cell ratio of 100:1), and the release of IL-10 (**a**) and IL-12p70 (**b**) and TNF-α (**c**) were assayed by ELISA. Results were represented as means ± SEM pg/mL for 9 independent healthy donors

We investigated the cytokine profiles stimulated by NS8 upon PBMCs collected from 9 independent donors. Although the absolute cytokine concentrations released by PBMCs varied between different donors, NS8 induced more significant increase of anti-inflammatory cytokine IL10, while less impact on release of proinflammatory cytokine IL12, in comparation with probiotic strain *L. casei* Shirota, which has been well characterized with health-promoting functions (Fig. 4). The ratio of IL10 to IL12 production was higher for NS8 (23.5) than *L. casei* Shirota (3.4). Both strains were able to induce TNF-α secretion from stimulated PBMCs, but the induction of this cytokine was partly lower when PBMCs were stimulated by NS8. These results indicated that IL-10 producing immune cells could evidently sense the stimulation of NS8.

#### Modulation the proinflammatory response triggered by LPS

**Fig. 5:**
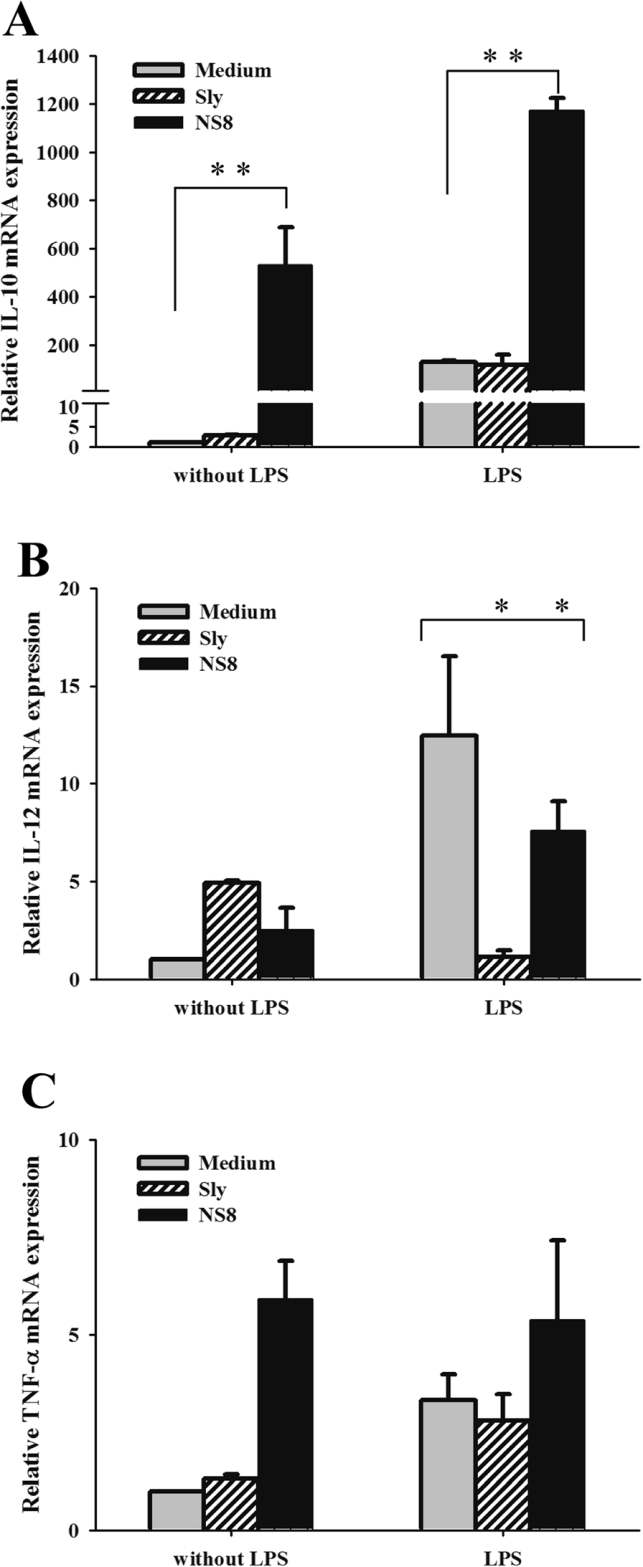
Modulation of the proinflammatory response triggered by LPS in RAW264.7 mouse macrophages. RAW264.7 cells were stimulated with *L. helveticus* NS8 (bacteria: cell ratio of 100:1) or its S-layer protein (10 μg/ml), with or without co-incubation of 1 μg/mL LPS for 4 h. Expression profiles of IL10 (**a**), IL-12p70 (**b**), and TNF-α (**c**) were examined by quantitative real-time PCR, indicated as the relative levels to the induction level of the control, which was set at a value of 1. Results are the means ± SD of representative of three independent experiments. Significant differences indicated as: **, *P* < 0.01; *, *P* < 0.05

In a final set of experiments, we tested the effects of NS8 on LPS-evoked inflammatory responses by analyzing the gene expression of two proinflammatory factors through qPCR, TNF-α and IL-12, and the anti-inflammatory cytokine IL-10 in mouse macrophage cell line RAW264.7. Here we also investigated whether the S-layer protein is involved in immunomodulatory activity exerted by NS8. After 4 h of co-stimulation of the RAW264.7 cells with LPS, NS8 induced a pronounced anti-inflammatory profile, as evidenced by an dramatically enhanced induction of IL-10 compared to the induction of TNF-α and IL-12 (Fig. 5). NS8 also decreased LPS-induced proinflammatory IL-12 production significantly (Fig. 5b). The reduction of IL-12 induced by purified S-layer protein was similar to that induced by NS8 strain itself, however, the expression of anti-inflammatory cytokine IL-10 was not influenced by S-layer protein (Fig. 5a, b). The results obtained by employing LPS-induced macrophages, therefore, confirmed the anti-inflammatory immune responses of NS8, but not dependent on S-layer protein.

## Discussion

As a traditionally fermented dairy product, koumiss has been getting more attentions for its healthy functions in recent years. The fermentation of Koumiss depends on the action of lactic acid bacteria and yeasts [11]. So in our study, we tried to select promising probiotic strains with particular health-promoting functions from Mongolian koumiss. After testing the tolerance ability to GIT environment and adherent potential to intestinal mucosa *in vitro*, we focused on a strain with good probiotic traits, *L. helveticus* NS8.

Comparative genomic studies have found that *L. helveticus* is rather closely related to the gut organism *L. acidophilus* although these two lactobacilli occupy different environmental niches [20]. However, *L. helveticus* DPC4571 was reported to possess a frame-shifted nonfunctional bile salt hydrolase gene, which suggested genetic adaption to the dairy environment might occur during the evolution [9]. That perhaps explained at least some part of the reason why NS8 showed moderate survival ability in the 0.3 % bile salt. Interestingly, in a recent study, *L. helveticus* was detected in every pasturing area Mongolian, but not in any of the city Mongolian, based on the 16S rRNA sequence analysis of human intestinal microbiota [21]. It suggested that dairy niche-specific bacteria, such as *L. helveticus*, still could survive and persist in human GIT under the condition of regular consumption. Nevertheless, for sustaining adequate populations of viable bacteria, we already developed enteric capsules of live NS8 bacteria.

When we tested the cellular surface properties of *L. helveticus* NS8, we observed an impressive adhesive capacity of NS8 to the human intestinal epithelial cells *in vitro*. Although some well adopted commercial probiotic strains showed adhesion as high as 15 % or as low as 2 %, the ability to adhere to the intestinal epithelium or mucosal surface had been considered as a major distinguishing feature for probiotic strains [22]. After adherence, probiotic bacteria should be able to autoaggregate and colonize in the gut for extending the interaction periods [23]. Compared with some other proposed probiotic strains, NS8 showed a significant autoaggregation in a very short time. That suggested the adherent cells of NS8 could potentially form a protective biofilm-like communities via autoaggregation or co-aggregation with commensal organisms on the intestinal mucosa. This close interaction with host intestinal mucosa might lead to the competitive exclusion of pathogens or the modulation of host cell responses [24].

Due to the importance of understanding immunological aspects when defining the probiotic potential, we mainly aimed to define whether *L. helveticus* NS8 could exert immunomodulatory effects to hosts. Firstly, in order to assess the anti-inflammatory potential of NS8, a standardized murine model of acute colitis induced by 2,4,6, trinitrobenzene sulphonic acid (TNBS) was applied by orally administered NS8 bacteria [25]. The prophylactic impact of NS8 strain was proven by observing that the weight loss and tissue damage were significantly alleviated. Recent increasing evidences indicated that both the systemic and mucosal immune systems can be modulated by orally delivered bacteria [26]. Foligne et al. proposed that *in vitro* IL-10/IL-12 cytokine induction ratio on PBMCs closely matched the ranking of the *in vivo* protective effect against TNBS-induced colitis in mice [16]. In agreement with the prevention *in vivo*, the IL-10/IL-12 ratio obtained after PBMCs stimulation confirmed a significant potential in the immunomodulatory properties of NS8. It is widely accepted that the health-promoting properties attributed to probiotics are multiple and strain-specific. Indeed, some *Lactobacillus* strains were shown as strong inducers of IL-12 and TNF-α, while in our study, NS8 was shown to be a strong inducer of IL-10. Similar immune responses could also be observed upon exposure to proinflammatory stimulus LPS, which is one of the best studied systemic inflammation inducer via simulation of Toll-like receptor4 pathway [27]. NS8 was able to reduce LPS-evoked inflammatory responses in mouse macrophage cell line RAW264.7 by inhibiting LPS-induced IL-12 production, while promoting higher levels of IL-10 in the presence of LPS compared to the levels induced by LPS alone. These results are important. IL-10 plays a central role in down-regulating inflammatory cascades and maintaining gut homeostasis [28]. Several studies also indicated that selective probiotics induce IL-10 production in the intestine or the development of IL-10-producing T cells *in vitro*, depending on the TLRs pathways [29, 30]. However, the precise mechanism by which specific strain induces IL-10-producing regulatory T cells remained unknown.

Specific probiotic characteristics of lactobacilli have been associated with the presence of particular surface molecules or structures, such as peptidoglycan, teichoic acids, exopolysaccharides and surface proteins, to evoke different host responses [31]. For instance, S-layer protein (SlpA) of *L. acidophilus* NCFM mediated the interaction of the bacteria with the ligand of the dendritic cell-specific intercellular adhesion molecule 3 (ICAM-3)-grabbing nonintegrin (DC-SIGN) and regulated DC immune functions [32]. S-layers are macromolecular paracrystalline arrays of proteins or glycoproteins commonly found in bacteria and archaea [33]. Some of these proteins were proven mediating the bacterium’s ability to antagonize pathogens, due to the capability of S-layer protein to efficiently adhere to the intestinal epithelium [33, 34]. As to NS8, absence of S-layer proteins promoted apparent morphology change of bacterial cells, suggesting that S-layer proteins are quite important for the surface property of NS8. Except that, purified SlpA protein of *L. acidophilus* NCFM was also responsible for the anti-inflammatory cytokine profile, because the protein induced higher levels of IL-10 in the presence of LPS [32]. However, our results suggested that the S-layer protein isolated from NS8 mainly attenuated LPS-induced IL-12 levels in macrophages, while didn’t influence the expression levels of IL-10. In contrast, the S-layer protein of *L. helveticus* MIMLh5 induced a proinflammatory effects in human U937 macrophages and macrophages isolated from mouse bone marrow (BMDMs) [10]. Such different data can be explained by considering the difference in surface proteins and different cell types used. The exact role of S-layer proteins in immune reactions remains elusive. To elucidate the molecular mechanisms determining the immunomodulatory capacity of NS8, the specific interactions between bacterial ligand and host receptor need to be learned in further study.

## Conclusion

In summary, NS8 isolate of *L. helveticus* from Mongolian fermented koumiss was identified as a good probiotic strain, based on the probiotic factors and immunomodulatory capacities. NS8 showed good hydrophobicity, cellular autoaggregation, cell adhesive capacity to enterocytes, and the presence of S-layer protein. Moreover, NS8-induced improvement in murine colitis is associated with the up-regulation of anti-inflammatory cytokine IL-10 in PBMCs and LPS-stimulated murine macrophage cell line RAW264.7. The present study indicates that NS8 can be useful in the development of nutraceutical products for the prevention or treatment of inflammation-associated diseases. Future studies should continue to address the mechanisms underlying the beneficial effects, taking into account the particular cell surface structures or metabolites of lactobacilli which influence the interaction with the host.

## Methods

### Genetic identification of lactobacilli isolates from koumiss

For isolation probiotic strains, home-made fermented koumiss were collected from Xilingol pastoral areas of Inner Mongolia. The milk samples were enriched in de Man, Rogosa and Sharpe (MRS) broth. Pour plating was also done with serial decimal dilutions and the submerged colonies were selected for morphological examination using Gram staining. The putative lactobacilli isolates were further subjected to catalase test and analysis of 16S rRNA gene sequences. The genomic DNA from the cultures was extracted by using DNA purification kit (TIANGEN, China). Genus specific PCR assays were carried out using two universal primers, 27f and 1492r [35]. The sequence data was aligned and analyzed using BLAST server available at NCBI website. Further confirmation of NS8 isolate was carried out by PCR using the *L. helveticus* specific primer pairs pepC/pepN/htrA [17] as well as primers targeted against *slpH* gene encoding S-layer protein (forward 5’GTTTAAGAATGGCAAGCG3’, reverse 5‘ACAAGAACAGCGACAAGC3’). Meantime, *L. acidophilus* specific primers targeted against *slpA* (forward 5‘GCTGGCTTTACTTGCTGTTGC3’, reverse 5‘CTCTTGCTTACGCTGGCTAC3’) were also applied into the PCR assay.

### Acid and bile salt tolerance

The resistance of lactobacilli isolates to gastrointestinal tract environment was tested as previously described [36]. Briefly, overnight cultures (10^7^–10^9^ CFU/mL) were harvested and washed twice with PBS buffer, before being resuspended in MRS broth with pH 2 or pH 3 or enriched with 0.2 or 0.3 % (w/v) Ox-Bile (Bio Basic Inc, China). After incubation at 37 °C for 2 h, acid tolerance was assessed in term of viable colony counts by MRS agar plating. For test of tolerance to bile salt, the cultures were inoculated for 4 h and colony counts were as well enumerated.

### Adhesion to Caco-2 Cells

Adhesion to Caco-2 cells was assayed as the method described by Jacobsen [37]. Caco-2 cells were grown in Dulbecco’s modified Eagle’s medium (DMEM, Hyclone, USA), supplemented with 10 % (v/v) fetal bovine serum (Gibco, USA), 100 U/mL penicillin, and 100 μg/mL streptomycin at 37 °C in a humidified 5 % CO_2_ atmosphere. Approximately 5 × 10^5^ Caco-2 cells were seeded in 6-well tissue culture plate and the medium was replaced by fresh nonsupplemented DMEM, at least 1 h before the bacterial suspension (c. 1 × 10^8^ CFU/mL) was added to each well. After co-incubation for 2 h, nonadherent cells were washed off by flushing with sterile PBS. Remaining adhered cells were detached by trypsinization and platted on MRS agar by serial dilution. Adhesion ability was expressed as the ratio between adherent bacteria and the initial number of added bacteria. Experiments were carried out in triplicate.

### Scanning electron microscopy

Scanning electron microscopy was also performed for qualitative examination of adhesion. Briefly, Caco-2 cells with adherent bacteria were fixed with 2.5 % glutaraldehyde for 24–72 h at 4 °C. The specimens were then dehydrated with a graded series of ethanol solutions (25, 50, 75, 90, and two times 100 %, 10 min each step). Critical-point drying and gold-coating were performed continuously, and specimens were then examined with a scanning electron microscope (Hitachi, S-3000 N).

### Cellular aggregation

Autoaggregation assays were performed by following the method of Del et al. [38]. The freshly grown bacterial cells were harvested and washed twice with PBS following resuspending again in PBS to get an absorbance of −0.5 at 600 nm (*A*_0_). During 5 h of incubation at room temperature, every hour 1 mL of the upper suspension was taken to measure the absorbance at 600 nm (*A*_t_). The autoaggregation percentage can be expressed as: (1 − *A*_t_/*A*_0_) × 100.

### Cell surface hydrophobicity

The bacterial surface hydrophobicity was determined according to the method of Rosenberg et al. [39]. Bacterial cells in the stationary phase were resuspended in 0.1 M KNO_3_ (pH 6.2) to approximately 10^8^ CFU/mL and the absorbance was measured at 600 nm (*A*_0_). One millilitre of xylene, chloroform or ethylacetate was separately mixed with 3 mL of bacterial suspension by vortexing and incubated at room temperature for 10 min. The mixture was again briefly vortexed and incubated at room temperature for 20 min for phase separations. The aqueous phase was gently moved to measure its absorbance at 600 nm (*A*_1_). The surface hydrophobicity (%) was calculated as (1 − *A*_1_/*A*_0_) × 100.

### Extraction of S-layer proteins

Extraction of the S-layer protein from *L. helveticus* was performed as described previously [40]. Washed cells were incubated with 5 M LiCl in a shaking incubator (200 rpm) for 60 min at 37 °C, followed by centrifugation at 10, 000 g, 4 °C for 10 min. The supernatant was dialyzed against distilled water at 4 °C for 48 h using a cellulose membrane with cut-off value of 10 kDa (Sigma). The extract was further concentrated by using Amicon Ultra (Millipore, USA). Protein concentration was measured by BCA protein assay before SDS-PAGE analysis.

### Induction of colitis *in vivo*

BALB/c mice (female, 8 weeks) purchased from Vital River Laboratories (China) were fed with regular rodent chow and tap water. After adaptation period, the mice were randomly selected and assigned to 3 groups of 10 mice each. For colitis induction, a standardized murine TNBS colitis model was used [25]. Briefly, anesthetized mice received an intrarectal administration of 40 μl solution of TNBS (100 mg/kg, Fluka) dissolved in 50 % ethanol. Control blank mice received 50 % ethanol. Bacterial suspensions (100 μL), containing 1 × 10^9^ CFU/mL in PBS buffer were administered intragastrically to mice each day, starting 5 days before until 1 day after TNBS processing. The mice were weighed and killed 48 h after TNBS administration. Colons were removed, washed and opened. Macroscopic lesions were evaluated according to the Wallace criteria [30]. Histological analysis was performed on hematoxylin/eosin-stained 5 μm tissue sections from colon samples fixed in 4 % paraformaldehyde and embedded in paraffin. The Animal Care and Ethics Committee at Hangzhou Normal University approved all of the animal experiments in our study.

### Isolation of peripheral mononuclear cells (PBMCs)

For isolation the PBMCs from human peripheral blood, nine blood samples of healthy donors were procured from Beijing Red Cross Blood Center. All the donors had written informed consent before donation. PBMCs were isolated as previously described [41]. Briefly, after a Ficoll gradient centrifugation, the buffy coat enriched with mononuclear cells was collected, washed in RPMI 1640 medium (Hyclone) and adjusted to 2 × 10^6^ cells/mL in RPMI 1640 supplemented with gentamicin (150 μg/mL), L-glutamine (2 mmol/L), and 10 % FBS.

### Cytokine induction and Elisa assay

PBMCs (1 × 10^6^ cells/mL) were seeded in 24-well tissue culture plates. Approximately 1 × 10^8^ CFU/mL lactobacilli were added (bacteria: cell ratio of 100:1) to different wells. After 24 h co-incubation, the culture medium was centrifuged at 12,000 g for 5 min at 4 °C, and the supernatant was collected and stored at −20 °C until cytokine measurement. Production of TNF-α, IL-10 and IL-12p70 in supernatants was measured by ELISA kit (BD Biosciences, USA) according to the manufacturer’s protocol.

### Macrophage culture and treatment

Mouse macrophage cell line RAW264.7 was used for studying inflammatory response. RAW264.7 cells were maintained in DMEM supplemented with 10 % FBS, 100 U/mL penicillin, and 100 μg/mL streptomycin at 37 °C. Approximately 5 × 10^5^ cells were seeded into 6-well tissue culture plate and allowed to adhere for 2 h prior to LPS activation. RAW264.7 cells were exposed to 1 μg/mL lipopolysaccharide (LPS, from *E. coli* serotype O127: B8, Sigma), followed by treated with lactobacilli (bacteria: cell ratio of 100:1) or 10 μg/mL S-layer protein purified from NS8 strain. The plates were incubated for 4 h before cytokine measurements.

### Real time PCR

Total RNA of RAW264.7 cells was extracted with TRIzol reagent (Invitrogen, USA). Reverse transcription was performed with a cDNA Reverse Transcription Kit (TIANGEN, China) according to the manufacturer’s instructions. Quantitative real-time PCR was carried out using Applied Biosystems 7300 (Life Technologies, USA). The reaction mixture was performed with SYBR® *Premix Ex Taq*™ (TaKaRa, China) according to the manufacturer’s protocols. The reaction conditions were 40 cycles of two-stage PCR consisting of denaturation at 95 °C for 15 s and annealing at 60 °C for 1 min after an initial denaturation step at 95 °C for 10 min The primer sequences were as follows: mouse TNF-α, forward 5‘ggcggtgcctatgtctcag3’ and reverse 5‘ggctacaggcttgtcactcg 3’; mouse IL-10, forward 5‘acatactgctaaccgactcct3’ and reverse 5‘ggtcttcagcttctcaccc3’; mouse IL-12p40, forward 5‘atgtggaatggcgtctc3’ and reverse 5‘gtctcctcggcagttgg3’; and mouse β-actin, forward 5‘agagggaaatcgtgcgtgac 3’ and reverse 5‘cgctcgttgccaatagtgat3’. For the relative comparison of mRNA expression levels, the data were analyzed with a ΔΔCt method and normalized to the amount of β-actin cDNA as an endogenous control.

### Statistical analysis

Data analysis was carried out with SPSS, Inc. software (version 10.0). Differences between two groups were assessed using the unpaired two-tailed Student’s *t*-test. Data sets that involved more than two groups were assessed using One-way ANOVA. Differences were considered significant if *P* was <0.05.

## Additional files

Additional file 1:
**Figure S1.** Autoaggregation percentage of *L. helveticus* NS8 in 5 hours. The sedimentation rate of strains was measured over a period of 5 h under identical conditions. NS8 isolate exhibited the highest cell autoaggregation. The autoaggregating phenotype of NS8 was so strong that over half bacterium have formed a precipitate in 1 h, while other bacterial suspensions showed constant turbidity with little precipitate. (PDF 159 kb) Additional file 2:
**Table S1.** Lewis acid-base characteristics of the bacterial cell surfaces. Adhesion to chloroform (electron acceptor) and ethyl acetate (electron donor) was also tested to assess the Lewis acid-base characteristics of the bacterial cell surfaces. All the strains didn’t show significant difference between the affinity to chloroform and to ethyl acetate. (PDF 46 kb)

## Abbreviations

BMDM: Bone marrow derived macrophage
DEME: Dulbecco’s modified Eagle’s medium
FBS: Fetal bovine serum
GIT: Gastrointestinal tract
IL: Interleukin
LPS: Lipopolysaccharide
MRS broth: De Man, Rogosa and Sharpe broth
PBMCs: Peripheral blood mononuclear cells
Slp: S-layer protein
TLR: Toll-like receptor
TNBS: 2,4,6-Trinitrobenzene sulfonic acid
TNF-α: Tumor necrosis factor alpha

## References

[1] Bergonzelli GE, Blum S, Brussow H, Corthesy-Theulaz I (2005). Probiotics as a treatment strategy for gastrointestinal diseases?. Digestion.

[2] Kumar M, Nagpal R, Kumar R, Hemalatha R, Verma V, Kumar A, et al. Cholesterol-lowering probiotics as potential biotherapeutics for metabolic diseases. Exp Diabesity Res. 2012;2012:902917. 10.1155/2012/902917 PMC3352670 22611376

[3] Ringel-Kulka T, Palsson OS, Maier D, Carroll I, Galanko JA, Leyer G, et al. Probiotic bacteria Lactobacillus acidophilus NCFM and Bifidobacterium lactis Bi-07 versus placebo for the symptoms of bloating in patients with functional bowel disorders: a double-blind study. J Clin Gastroenterol. 2011;45(6):518–25. 10.1097/MCG.0b013e31820ca4d6 PMC4372813 21436726

[4] Szajewska H, Wanke M, Patro B (2011). Meta-analysis: the effects of Lactobacillus rhamnosus GG supplementation for the prevention of healthcare-associated diarrhoea in children. Aliment Pharmacol Ther.

[5] Heller KJ (2001). Probiotic bacteria in fermented foods: product characteristics and starter organisms. Am J Clin Nutr.

[6] Fortina MG, Nicastro G, Carminati D, Neviani E, Manachini PL (1998). Lactobacillus helveticus heterogeneity in natural cheese starters: the diversity in phenotypic characteristics. J Appl Microbiol.

[7] Callanan M, Kaleta P, O’Callaghan J, O’Sullivan O, Jordan K, McAuliffe O, et al. Genome sequence of Lactobacillus helveticus, an organism distinguished by selective gene loss and insertion sequence element expansion. J Bacteriol. 2008;190(2):727–35. 10.1128/JB.01295-07 PMC2223680 17993529

[8] Rachid M, Matar C, Duarte J, Perdigon G (2006). Effect of milk fermented with a Lactobacillus helveticus R389(+) proteolytic strain on the immune system and on the growth of 4 T1 breast cancer cells in mice. FEMS Immunol Med Microbiol.

[9] Slattery L, O’Callaghan J, Fitzgerald GF, Beresford T, Ross RP (2010). Invited review: Lactobacillus helveticus--a thermophilic dairy starter related to gut bacteria. J Dairy Sci.

[10] Taverniti V, Stuknyte M, Minuzzo M, Arioli S, De Noni I, Scabiosi C, et al. S-layer protein mediates the stimulatory effect of Lactobacillus helveticus MIMLh5 on innate immunity. Appl Environ Microbiol. 2013;79(4):1221–31. 10.1128/AEM.03056-12 PMC3568609 23220964

[11] Mu Z, Yang X, Yuan H (2012). Detection and identification of wild yeast in Koumiss. Food Microbiol.

[12] Jagielski VA (1874). On the Various Preparations of Koumiss, and their Use in Medicine. Br Med J.

[13] Tsenina VS, Frolov VM (1980). Use of koumiss made from cow’s milk in liver diseases. Meditsinskaia sestra.

[14] Christensen HR, Frokiaer H, Pestka JJ (2002). Lactobacilli differentially modulate expression of cytokines and maturation surface markers in murine dendritic cells. J Immunol.

[15] Vitini E, Alvarez S, Medina M, Medici M, de Budeguer MV, Perdigon G (2000). Gut mucosal immunostimulation by lactic acid bacteria. Biocell : Off J Sociedades Latinoamericanas de Microscopia Electronica et al.

[16] Foligne B, Nutten S, Grangette C, Dennin V, Goudercourt D, Poiret S, Dewulf J, Brassart D, Mercenier A, Pot B (2007). Correlation between *in vitro* and *in vivo* immunomodulatory properties of lactic acid bacteria. WJG.

[17] Fortina MG, Ricci G, Mora D, Parini C, Manachini PL (2001). Specific identification of Lactobacillus helveticus by PCR with pepC, pepN and htrA targeted primers. FEMS Microbiol Lett.

[18] Chen K, Liang N, Luo X, Zhang TC (2013). Lactobacillus acidophilus strain suppresses the transcription of proinflammatory-related factors in human HT-29 cells. J Microbiol Biotechnol.

[19] Gu XC, Luo XG, Wang CX, Ma DY, Wang Y, He YY, et al. Cloning and analysis of bile salt hydrolase genes from Lactobacillus plantarum CGMCC No. 8198. Biotechnol Lett. 2013. doi:10.1007/s10529-013-1434-9. 10.1007/s10529-013-1434-9 24375235

[20] Wasko A, Polak-Berecka M, Kuzdralinski A, Skrzypek T (2014). Variability of S-layer proteins in Lactobacillus helveticus strains. Anaerobe.

[21] Zhang J, Zheng Y, Guo Z, Qiao J, Gesudu Q, Sun Z, et al. The diversity of intestinal microbiota of Mongolians living in Inner Mongolia, China. Benefic Microbes. 2013;4(4):319–28. 10.3920/BM2013.0028 24311315

[22] Tuomola EM, Salminen SJ (1998). Adhesion of some probiotic and dairy Lactobacillus strains to Caco-2 cell cultures. Int J Food Microbiol.

[23] Collado M, Meriluoto J, Salminen S (2008). Adhesion and aggregation properties of probiotic and pathogen strains. Eur Food Res Technol.

[24] Velez MP, De Keersmaecker SC, Vanderleyden J (2007). Adherence factors of Lactobacillus in the human gastrointestinal tract. FEMS Microbiol Lett.

[25] Foligne B, Nutten S, Steidler L, Dennin V, Goudercourt D, Mercenier A, et al. Recommendations for improved use of the murine TNBS-induced colitis model in evaluating anti-inflammatory properties of lactic acid bacteria: technical and microbiological aspects. Dig Dis Sci. 2006;51(2):390–400. 10.1007/s10620-006-3143-x 16534687

[26] Sheil B, McCarthy J, O’Mahony L, Bennett MW, Ryan P, Fitzgibbon JJ, et al. Is the mucosal route of administration essential for probiotic function? Subcutaneous administration is associated with attenuation of murine colitis and arthritis. Gut. 2004;53(5):694–700. 10.1136/gut.2003.027789 PMC1774028 15082588

[27] Lu Y-C, Yeh W-C, Ohashi PS (2008). LPS/TLR4 signal transduction pathway. Cytokine.

[28] Mohamadzadeh M, Olson S, Kalina WV, Ruthel G, Demmin GL, Warfield KL, et al. Lactobacilli activate human dendritic cells that skew T cells toward T helper 1 polarization. Proc Natl Acad Sci U S A. 2005;102(8):2880–5. 10.1073/pnas.0500098102 PMC549474 15710900

[29] Jeon SG, Kayama H, Ueda Y, Takahashi T, Asahara T, Tsuji H, et al. Probiotic Bifidobacterium breve induces IL-10-producing Tr1 cells in the colon. PLoS Pathog. 2012;8(5), e1002714. 10.1371/journal.ppat.1002714 PMC3364948 22693446

[30] Rachmilewitz D, Katakura K, Karmeli F, Hayashi T, Reinus C, Rudensky B, et al. Toll-like receptor 9 signaling mediates the anti-inflammatory effects of probiotics in murine experimental colitis. Gastroenterology. 2004;126(2):520–8. 10.1053/j.gastro.2003.11.019 14762789

[31] Lebeer S, Vanderleyden J, De Keersmaecker SC (2008). Genes and molecules of lactobacilli supporting probiotic action. MMBR.

[32] Konstantinov SR, Smidt H, de Vos WM, Bruijns SC, Singh SK, Valence F, et al. S layer protein A of Lactobacillus acidophilus NCFM regulates immature dendritic cell and T cell functions. Proc Natl Acad Sci U S A. 2008;105(49):19474–9. 10.1073/pnas.0810305105 PMC2592362 19047644

[33] Johnson-Henry KC, Hagen KE, Gordonpour M, Tompkins TA, Sherman PM (2007). Surface-layer protein extracts from Lactobacillus helveticus inhibit enterohaemorrhagic Escherichia coli O157:H7 adhesion to epithelial cells. Cell Microbiol.

[34] Chen X, Xu J, Shuai J, Chen J, Zhang Z, Fang W (2007). The S-layer proteins of Lactobacillus crispatus strain ZJ001 is responsible for competitive exclusion against Escherichia coli O157:H7 and Salmonella typhimurium. Int J Food Microbiol.

[35] Ludwig W (2007). Nucleic acid techniques in bacterial systematics and identification. Int J Food Microbiol.

[36] Conway PL, Gorbach SL, Goldin BR (1987). Survival of lactic acid bacteria in the human stomach and adhesion to intestinal cells. J Dairy Sci.

[37] Jacobsen CN, Rosenfeldt Nielsen V, Hayford AE, Moller PL, Michaelsen KF, Paerregaard A, et al. Screening of probiotic activities of forty-seven strains of Lactobacillus spp. by *in vitro* techniques and evaluation of the colonization ability of five selected strains in humans. Appl Environ Microbiol. 1999;65(11):4949–56. 10.1128/aem.65.11.4949-4956.1999 PMC91666 10543808

[38] Del Re B, Sgorbati B, Miglioli M, Palenzona D (2000). Adhesion, autoaggregation and hydrophobicity of 13 strains of Bifidobacterium longum. Lett Appl Microbiol.

[39] Rosenberg M, Gutnick D, Rosenberg E (1980). Adherence of bacteria to hydrocarbons: A simple method for measuring cell-surface hydrophobicity. FEMS Microbiol Lett.

[40] Lortal S, Van Heijenoort J, Gruber K, Sleytr UB (1992). S-layer of Lactobacillus helveticus ATCC 12046: isolation, chemical characterization and re-formation after extraction with lithium chloride. J Gen Microbiol.

[41] Grangette C, Nutten S, Palumbo E, Morath S, Hermann C, Dewulf J, et al. Enhanced antiinflammatory capacity of a Lactobacillus plantarum mutant synthesizing modified teichoic acids. Proc Natl Acad Sci U S A. 2005;102(29):10321–6. 10.1073/pnas.0504084102 PMC1177390 15985548

