# Supplementary material for: Probiotic and anti-inflammatory attributes of an isolate Lactobacillus helveticus NS8 from Mongolian fermented koumiss
Source: BMC Microbiol. 2015 Oct 2;15:196. doi: 10.1186/s12866-015-0525-2 (PMC4591576; doi:10.1186/s12866-015-0525-2)
Supplement: Additional file 1: — Figure S1. Autoaggregation percentage of L. helveticus NS8 in 5 hours. The sedimentation rate of strains was measured over a period of 5 h under identical conditions. NS8 isolate exhibited the highest cell autoaggregation. The autoaggregating phenotype of NS8 was so strong that over half bacterium have formed a precipitate in 1 h, while other bacterial suspensions showed constant turbidity with little precipitate. (PDF 159 kb) [file 12866_2015_525_MOESM1_ESM.pdf]

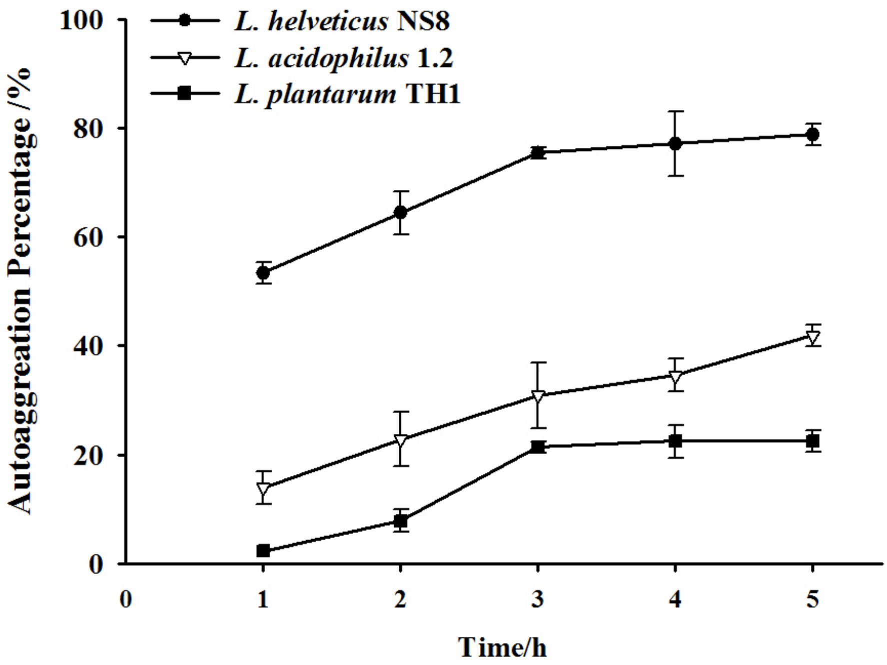

Figure. Autoaggregation percentage of *L. helveticus* NS8 in 5 hours.

The sedimentation rate of strains was measured over a period of 5 h under identical conditions. NS8 isolate exhibited the highest cell autoaggregation. The autoaggregating phenotype of NS8 was so strong that over half bacterium have formed a precipitate in 1 h, while other bacterial suspensions showed constant turbidity with little precipitate.
