# Supplementary material for: Probiotic and anti-inflammatory attributes of an isolate Lactobacillus helveticus NS8 from Mongolian fermented koumiss
Source: BMC Microbiol. 2015 Oct 2;15:196. doi: 10.1186/s12866-015-0525-2 (PMC4591576; doi:10.1186/s12866-015-0525-2)
Supplement: Additional file 2: — Table S1. Lewis acid-base characteristics of the bacterial cell surfaces. Adhesion to chloroform (electron acceptor) and ethyl acetate (electron donor) was also tested to assess the Lewis acid-base characteristics of the bacterial cell surfaces. All the strains didn’t show significant difference between the affinity to chloroform and to ethyl acetate. (PDF 46 kb) [file 12866_2015_525_MOESM2_ESM.pdf]

Table. Lewis acid-base characteristics of the bacterial cell surfaces

Bacteria	Hydrophobicity (%)	
	**Chloroform**	**Ethyl acetate**
*L. helveticus*NS8	53.1 ± 0.8	60.8 ± 3.9
L. acidophilus 1.2	69.2 ± 1.3	74.1 ± 0.3
L. plantarum TH1	55.1 ± 1.5	64.5 ± 0.9

Adhesion to chloroform (electron acceptor) and ethyl acetate (electron donor) was also tested to assess the Lewis acid-base characteristics of the bacterial cell surfaces. All the strains didn’t show significant difference between the affinity to chloroform and to ethyl acetate.
